# Systematic Review with Meta-Analysis: Effectiveness and Safety of Acupuncture as Adjuvant Therapy for Side Effects Management in Drug Therapy-Receiving Breast Cancer Patients

**DOI:** 10.1155/2021/9949777

**Published:** 2021-10-12

**Authors:** Yau-Tuen Chan, Ning Wang, Chi-Wing Tam, Hor-Yue Tan, Yuanjun Lu, Tsz-him So, Edwin Chau-Leung Yu, Lixing Lao, Yibin Feng

**Affiliations:** ^1^School of Chinese Medicine, Li Ka Shing Faculty of Medicine, The University of Hong Kong, Hong Kong, China; ^2^Department of Clinical Oncology, Li Ka Shing Faculty of Medicine, The University of Hong Kong, Hong Kong, China; ^3^Hong Kong Association for Integration of Chinese-Western Medicine, Hong Kong, China; ^4^Virginia University of Integrative Medicine, 9401 Mathy Dr, Fairfax, VA 22031, USA

## Abstract

**Objective:**

To investigate the potential benefits and safety of acupuncture on managing side effects induced by drug therapies in patients with breast cancer using a PRISMA standard systematic review and meta-analysis.

**Methods:**

Published randomised controlled trials from nine databases in English and Chinese language were searched. Trials with a real acupuncture treatment group and a control group with sham acupuncture, no treatment, or waitlist control were included. The primary outcome of this study was the therapeutic effects on five symptoms induced by drug therapies, including gastrointestinal disorder, neuropathy, arthralgia, joint symptoms, and cognitive impairment. The quality of life was assessed as a secondary outcome. The risk of bias of each study was analysed according to the Cochrane Handbook.

**Results:**

Sixteen randomised controlled trials with 1189 participants were included in the meta-analysis. The primary outcome and all subgroup analyses showed statistically significant improvements in the management of side effects by real acupuncture. The quality of life of patients has enhanced during the treatment.

**Conclusion:**

Although the number of publications is limited, a clear preliminary conclusion could be drawn by the meta-analysis, suggesting the beneficial adjuvant role of acupuncture in patients with breast cancer who receive drug therapies. No serious adverse events were observed from all the RCTs, and the safety of acupuncture is ascertained. More standardised and sophisticated large-scale randomised controlled trials are needed to evaluate the findings further.

## 1. Background

Breast cancer is the fifth leading cause of cancer mortality worldwide [1]. Current treatment strategies include surgical excision, hormonal therapy, radiation therapy, chemotherapy, and antibody treatment [2]. Adjuvant treatments are often offered to patients after mastectomy. However, side effects are commonly observed from the patients, especially receiving drug therapy regimens. Fatigue, hair loss, nausea, vomiting, loss of appetite, and diarrhoea are some of the milder side effects, while, in severe cases, it could lead to infertility, arthralgia, neuropathy, and cognitive impairments [3, 4]. Relieving the side effects of drug therapy is essential and beneficial to cancer patients.

Acupuncture has been used for thousands of years in the traditional Chinese medicine practice. It is suggested that acupuncture may have appeared earlier than herbal medicine. It is widely used in western countries too as an alternative medicine to treat headaches, migraines, pain, osteoarthritis, and certain respiratory disorders [5]. It is also useful in reducing the side effects induced by drug therapies. Several systemic reviews had discussed some aspects of cancer treatment side effects by acupuncture, but recent updates are unavailable on the specific benefits of acupuncture to the breast cancer patients receiving drug therapy. Pan et al. studied the clinical benefits of acupuncture on hormone therapy-related side effects in breast cancer [6], while the study by Roberts et al. included adjuvant treatments other than acupuncture [7]. A systematic review researching the beneficial effects of herbal medicine on side-effect management is available [8]; however, a comprehensive meta-analysis of using acupuncture for managing the side effects induced by drug therapies is still deficient. Acupuncture has a systematic approach that could enhance the body conditions and relieve symptoms as a whole; therefore, most of the case studies included multiple side-effect managements.

In this study, a systematic review and meta-analysis were performed according to the PRISMA statement [9]. This review focuses on five side effects induced by drug therapy in patients with breast cancer, regardless of the stage of the disease. The five side effects are as follows: gastrointestinal disorders, chemotherapy-induced peripheral neuropathy (CIPN), aromatase inhibitor-associated arthralgia (AIAA), aromatase inhibitor-associated joint problems (AIAS), and cognitive impairment. Randomised controlled clinical trials published in English and Chinese language were analysed. The included studies were published from 2000 to 2020. The effects on symptom management were the primary outcome, while the effects on quality of life (QoL) were the secondary outcome. The symptoms were also separately studied in the subgroup analysis.

## 2. Methods

The study methodology of this systematic review was designed according to the PRISMA practice [9]. The study protocol has been registered on the PROSPERO database by NIHR with the ID CRD42020187399.

### 2.1. Search Strategies

Published randomised controlled trials (RCTs) reports were searched on central electronic databases from their inception until the present, including Cochrane Library (1996–2020), Web of Science (1956–2020), EMBASE (1947–2020), MEDLINE (1946–2020), Pubmed (1966–2020), CINAHL Plus (1937–2020), AMED (1985–2020), CNKI (1911–2020), and Wanfang Data (1989–2020). References from related systematic reviews were reviewed and checked for potential inclusion. Unpublished data were not included.

The corresponding detailed search syntax for each database was listed in Supplementary Table 2. The search strategies were adjusted in various English and Chinese databases to suit the different language style and database instructions.

### 2.2. Study Selection

#### 2.2.1. Type of Studies

Only RCTs studying the effect of acupuncture on relieving the side effects of drug therapies that treat breast cancer were included. Both blinded and unblinded RCTs were included to increase the sample size and were assessed using the risk of bias table. Incomplete studies and unpublished data were not included. Studies with sample sizes of less than ten were not included.

#### 2.2.2. Participants

Participants had breast cancer regardless of the stage of cancer. They must have received or been receiving any kinds of drug therapies before or during the treatment period of the study. All ages, races, and origins were included.

#### 2.2.3. Intervention and Inclusion/Exclusion

Acupuncture treatment was the only intervention in the treatment group. The control group received sham acupuncture or no treatment, or the participants were included as waitlist control group. To reduce the heterogeneity, only interventions with penetrating needles on the acupoints were included. Other methods of stimulation like moxibustion, laser-stimuli, massage, and acupressure were excluded.

#### 2.2.4. Outcome Measures

Primary outcomes were standard mean differences (SMD) of side effect level indices between the experimental group (real acupuncture) and control group (sham acupuncture/no treatment/waitlist control). Only the following side effects were included in these studies: drug therapy-induced gastrointestinal disorders, chemotherapy-induced peripheral neuropathy, aromatase inhibitor-associated arthralgia, aromatase inhibitor-associated joint symptoms, and cognitive impairment. The measure of the quality of life was the secondary outcome measurement. Effects on physical, social, emotional and mental well-being were included in the measurement of QoL.

#### 2.2.5. Data Extraction and Study Bias Assessment

The search results were imported into the Endnote X8. Two authors independently screened the results through titles and abstracts and assessed the eligibility by reading the full text according to the selection criteria. Data were extracted independently in duplicate using a detailed structured form. Risk of bias was evaluated afterwards using the Cochrane standard. There were in total six categories in the risk management table, namely, “random sequence generation,” “allocation concealment,” “blinding of participants and personnel,” “blinding of outcome assessment,” “incomplete outcome data” and “selective reporting.” Each category was rated with low risk, unclear risk, or high risk. For the “random sequence generation,” any means of randomisation such as computer software or random number table would be considered appropriate and rated as “low risk”; an example of “low risk” allocation process is concealing the patient assignment in opaque sealed envelopes. For blinding, if patients or investigators were blinded to the group assortment, they would be rated as “low risk.” Only patients receiving sham control could be considered as low risk, while no treatment and waitlist control would make the patient side unblinded. It would be a low risk of detection bias when the statisticians or investigators were blinded to the identities of the patients, even if the acupuncturists knew the treatment. Finally, attrition bias measures the proportion of patients dropping out before the primary outcome was measured during the treatment. According to the Cochrane Handbook 8.5.2, a proportion of less than 5% is rated as “low,” while a proportion larger than 20% is rated as “high” [10]. If only a small proportion of patients dropped out with a detailed record of reason, “low risk” would be rated in reporting bias. Otherwise, unclear or unexplained dropping out would make be an “unclear or high risk.” A total of three or more “low risk” ratings would classify that study to be of high quality. The randomised trials were also assessed using the Grading of Recommendations, Assessment, Development, and Evaluation (GRADE) guidelines. The certainty of evidence from RCTs were initially regarded as “high ⊕⊕⊕⊕,” and were downgraded to “moderate ⊕⊕⊕○ “low ⊕⊕○○” and “very low ⊕○○○” quality, depending on the following five criteria: risk of bias (limitations in the study design and implementation), indirectness (of evidence), inconsistency (high and unexplained heterogeneity of results), imprecision (of results), and (high probability of) publication bias.

#### 2.2.6. Statistical Analysis

Statistical analysis was performed using the Review Manager 5.3 for Windows (The Nordic Cochrane Centre, Copenhagen, Denmark). Mean changes of the indices were normalised to a range of “−1” to “1” using the maximum score in the corresponding scale. “1” is the largest increasing proportion, and “−1” is total reduction of the measurement (e.g., a reduction in pain level score would result in a negative mean change). Continuous outcomes are analysed by standard mean difference in the inverse variance random-effect model. For those studies not providing standard deviation, 95% confidence interval (CI) was converted to standard deviation (SD) by using the formula SD = sqrt(*n*)∗(95% CI)/3.92 [11]. Therapeutic effects were included regardless of the acupoints and methods of electrostimulations. Heterogeneity was observed by forest plot and calculated by the Review Manager software and presented as *I*^2^ value, where 25%, 50%, and 75% were regarded as low, moderate, and high heterogeneity. When high heterogeneity was achieved, sensitivity analysis was performed to check the potential presence of outlier studies. Subgroup analysis was carried out by different side effect symptoms of drug therapy. *p* value <0.05 was considered as statistically significant.

## 3. Results

### 3.1. Study Characteristics

**Figure 1:**
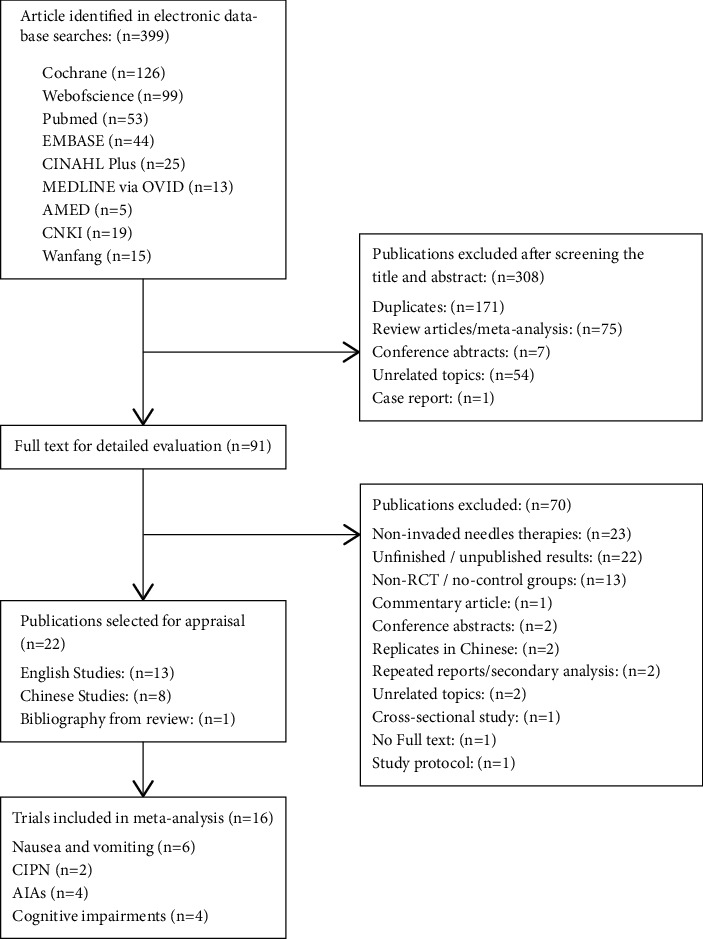
Flow diagram of study selection of this systematic review.

The progress of screening and selecting trials to the meta-analysis was shown in Figure 1. After the first search from the databases, 399 results were obtained. After the evaluation of the title, abstract, and full text, 21 published RCTs were selected for appraisal. Thirteen of them were English studies, and eight of them were from Chinese language journals or thesis. During the meta-analysis, six additional publications were excluded from the studies, but one extra RCT was included from the bibliography of a systematic review [12]. Sixteen RCTs were included in the final meta-analysis [12–27]. In two of the excluded studies, there was no information about the measurement of the patient response but only positive result percentage was shown. Another study was excluded because the treatment period was inconsistent in all patients. Other exclusion reasons included lack of a comparable control group, replicated publishing results in journal article/thesis, and incomparable measurement. Ten studies were published in English and six in Chinese in the years 2000 to 2020. A total of 1230 patients (645: real acupuncture arm; 585: control arm) were included in this meta-analysis. The sample sizes of the groups ranged from 15 to 101. The detailed characteristics of the included studies are summarised in Table 1.

### 3.2. Study Quality Assessment

**Figure 2:**
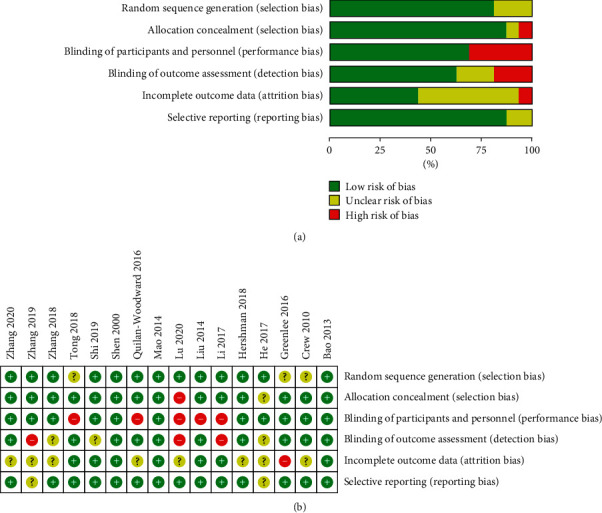
Risk of bias assessment of the randomised trials. (a) Risk of bias graph. (b) Risk of bias summary table.

The 16 included studies were assessed with the risk of bias table. More than half of the low-bias-risk studies existed in most of the categories, except the attrition bias. The higher bias in few of the studies was due to a high dropping-out rate or loss to follow-up. Five of the 16 studies were not patient-blinded trials or without sham control, while six of them had unblinded investigators. All the included trials were randomised, from which 13 mentioned the methods of randomisation and allocation. Fourteen out of the sixteen studies provided reasons for withdrawals or had no withdrawals at all. The overall risk of bias was shown in Figure 2; all but two studies (He 2017 and Lu 2020) were of high quality with low risk of bias. Every included study had passed the quality assessment. The level of evidence was also assessed using the GRADE guideline, and the results are listed in Table 2.

### 3.3. Primary Outcomes

**Figure 3:**
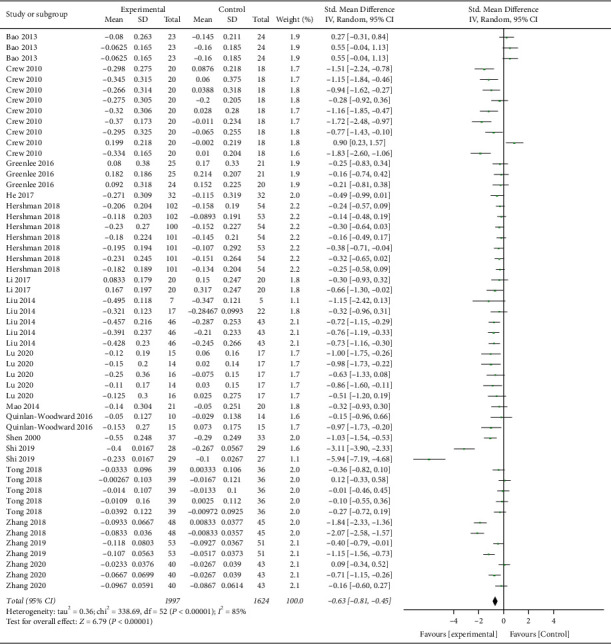
Forest plot of studies comparing the adjuvant effects of real acupuncture and control treatment: primary outcome.

Fifty-three measurements from all 16 studies were included in the primary outcome analysis (Figure 3). The total participants from the treatment arm and control arm were 1997 and 1624 patients, respectively. All the results from measurements were normalised into a “−1” to “1” scale, where “−1” was the maximum score favouring real acupuncture side and “1” was favouring control side in each set. A negative mean change represented an improvement, while a negative SMD favoured acupuncture side. The pooled SMD was −0.63 (95% CI −0.81, −0.45; *p* < 0.00001), which signified a strong effect favouring the real acupuncture side. Nearly all measurement has a favoured value on the real acupuncture arm, which presented a very consistent result. Acupuncture could alleviate the side effects brought about by drug therapy that treat breast cancer to a certain extent.

### 3.4. Subgroup Analysis

To further illustrate the adjuvant effects of acupuncture in managing the side effects, subgroup analysis was performed according to the symptoms caused by drug therapies. Five subgroup analyses were performed, namely, gastrointestinal disorders, chemotherapy-induced peripheral neuropathy, aromatase inhibitor-associated arthralgia, aromatase inhibitor-associated joint symptoms, and cognitive impairment.

#### 3.4.1. Gastrointestinal Disorders

**Figure 4:**
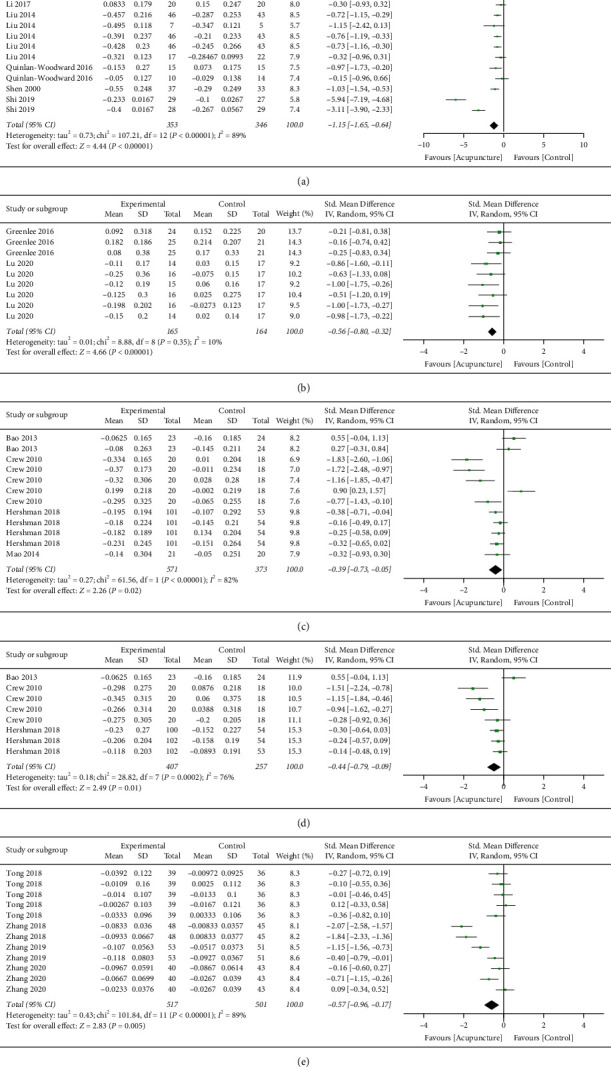
Forest plot of subgroup analysis. (a) Gastrointestinal disorders, (b) chemotherapy-induced peripheral neuropathy, (c) aromatase inhibitor-associated arthralgia, (d) aromatase inhibitor-associated joint symptoms, and (e) cognitive impairment.

Six trials reported nausea and vomiting [13, 14, 17, 19, 20, 24]. Thirteen measurements from the trials were included in the subgroup analysis, where 353 patients were in the treatment arm and 346 patients were in the control arm. Acupuncture was suggested to be superior to control treatment in controlling nausea and vomiting induced by drug therapy treatment (pooled SMD = −1.15; 95% CI (−1.65, −0.64); *p* < 0.00001; *I*^2^ = 89%). All measurements in this category favoured the treatment side (Figure 4(a)). The gastrointestinal disorders measurements involved in this analysis were nausea level, vomiting level, appetite level, constipation score, diarrhoea score, and emesis episodes. Acupuncture showed beneficial effects in all of them as suggested by the unanimous result.

#### 3.4.2. Chemotherapy-Induced Peripheral Neuropathy

Only two published trials could fit the selection criteria of this systematic review after screening [18, 26]. From the two trials, a total of nine measurements were related to CIPN and pain level scores (Figure 4(b)). There were 165 patients in the treatment arm and 164 patients in the control arm. This subgroup analysis supported that acupuncture could effectively reduce CIPN in patients with breast cancer (pooled SMD = -0.56; 95% CI (−0.80, −0.32); *p* < 0.00001; *I*^2^ = 10%). The pain level indices included in this analysis were BPI-SF worst pain, BPI-SF pain severity, BPI-SF pain interference, FACT-NTX summary score, NPS-4, PNQ sensory score, and PNQ motor score. Acupuncture showed beneficial effects in all of them as suggested by the unanimous result.

#### 3.4.3. Aromatase Inhibitor-Associated Arthralgia (AIAA)

Four studies focused on aromatase inhibitor-associated symptoms [12, 15, 16, 21]. The symptoms were separated into two subgroup analyses, arthralgia, and other joint symptoms.

For the AIAA, there were 12 measurements involved in this analysis from the four trials. A total of 571 and 373 patients were recorded in the acupuncture and control arm, respectively. A significant effect by acupuncture was observed (pooled SMD = −0.39; 95% CI (−0.73, −0.05); *p*=0.02; *I*^2^ = 82%). However, three measurements reported having an effect favouring the control treatment side, two of which were from the same study (Figure 4(c)). The pain level indices involved in this subgroup analysis of AIAA included HAQ-DI, pain VAS score, BPI-WP, BPI pain severity, BPI pain-related interference, BPI average pain, WOMAC pain, and M-SACRAH pain. Also, by having a slightly inconsistent result, the overall negative SMD with *p* value 0.02 proves that acupuncture has beneficial effects on reducing AIAA over control treatment.

#### 3.4.4. Aromatase Inhibitor-Associated Joint Symptoms (AIAS)

The AIAS subgroup in the present study includes joint function and joint stiffness that can affect the motor ability of the patients. Three trials provided eight measurements in this subgroup analysis [15, 16, 21] (Figure 4(d)). Four hundred and seven patients were included in the acupuncture arm, while 257 patients were included in the control arm. Acupuncture was found to be more beneficial to the patients than the control treatment in this area (pooled SMD = −0.44; 95% CI (−0.79, −0.09); *p* = 0.01; *I*^2^ = 76%). The measurement indices involved in this subgroup analysis included HAQ-DI, WOMAC stiffness, WOMAC function, M-SACRAH stiffness, M-SACRAH function, and BPI worst stiffness.

#### 3.4.5. Cognitive Impairment

Four trials reporting the acupuncture treatment on cognitive impairment induced by drug therapy were included in this subgroup analysis after screening [22, 23, 25, 27]. From the 12 measurements, 517 patients were included in the acupuncture arm and 501 patients in the control arm (Figure 4(e)). A confident therapeutic effect was observed again by the acupuncture treatment (pooled SMD = −0.57; 95% CI (−0.96, −0.17); *p* = 0.005; *I*^2^ = 89%). The measurements involved in this subgroup analysis were FACT-COG, AVLT, MoCA, MMSE, and forward and reverse digit span test.

### 3.5. Sensitivity Analysis

**Figure 5:**
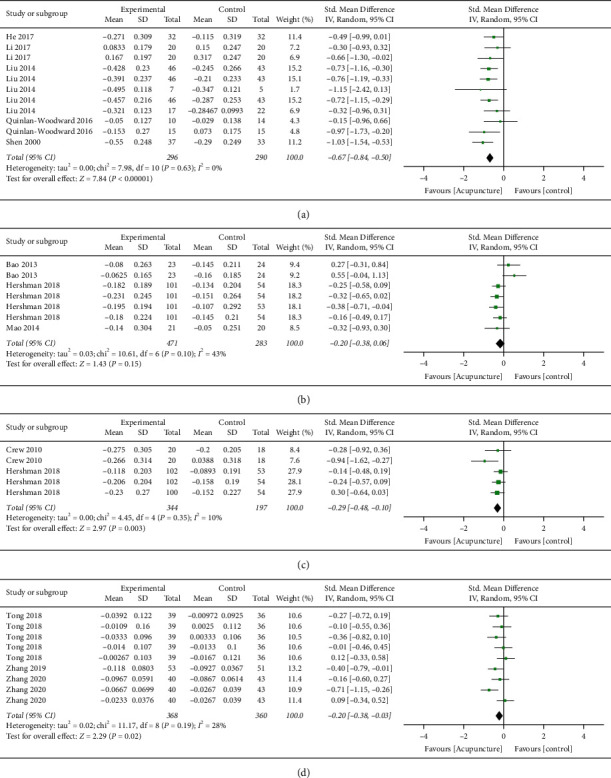
Forest plots of the sensitivity analysis on subgroups. (a) Gastrointestinal disorders, (b) aromatase inhibitor-associated arthralgia, (c) aromatase inhibitor-associated joint symptoms, and (d) cognitive impairment.

From the subgroup sensitivity analyses, the effect size of acupuncture on gastrointestinal disorders was the greatest and prominently larger than the average primary outcome (pooled SMD = −1.15 vs −0.63). The protruding effect of this group was neutralized when the result from a single trial [24] was excluded in the subgroup, which heterogeneity becomes perfectly low (pooled SMD = −0.67; 95% CI (−0.84, −0.5); *p* < 0.00001; *I*^2^ = 0%) (Figure 5(a)). For the sensitivity analyses in the AIAA subgroup, one study [15] showed an impactful effect on both the outcome and heterogeneity. After excluding this study, the meta-analysis showed a nonstatistically significant minimal effect and medium heterogeneity (pooled SMD = −0.15; 95% CI (−0.36, 0.06); *p* = 0.15; *I*^2^ = 43%) (Figure 5(b)). Sensitivity analysis yielded three outcomes from two trials [15, 16] with outstanding heterogeneity for the subgroup analysis on AIAS. The directional effect remained but reduced after excluding these results, and the heterogeneity was becoming low (pooled SMD = −0.29; 95% CI (−0.48, −0.10); *p* = 0.003; *I*^2^ = 10%) (Figure 5(c)). The final sensitivity analysis was performed onto the cognitive impairment subgroup, where three results from two trials [23, 25] were excluded. The effect sizes and heterogeneity reduced substantially, but the statistics was still significant (pooled SMD = −0.20; 95% CI (−0.38, −0.03); *p* = 0.02; *I*^2^ = 28%) (Figure 5(d)).

### 3.6. Secondary Outcome

**Figure 6:**
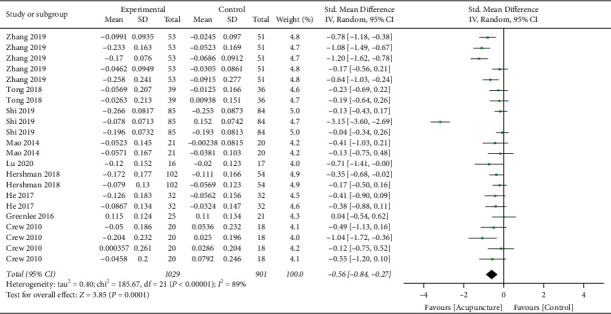
Forest plot of secondary outcome: quality of life analysis.

To further investigate the additional benefits brought about by acupuncture, a secondary outcome measuring the QoL of patients was used as an indicator (Figure 6). Out of the 16 screened studies, nine of them reported measurements related to the study of QoL [12, 13, 15, 18, 21, 22, 24–26]. Twenty-two measurements involving 1029 and 901 patients from the acupuncture and control arms were included in the secondary analysis. A homogeneous result favouring the acupuncture side was observed (pooled SMD = −0.56; 95% CI (−0.84, −0.27); *p* = 0.0001; *I*^2^ = 89%). The measurements involved in the secondary outcome related to QoL were FACT-G, FACT-TAX, FACT-ES, FACT-COG, HADS, PROMIS PI-SF, SAS, SDS, KPS, and EORTC QLQ-C30. The real acupuncture was shown to have both physical and mental benefits on patients with breast cancer.

## 4. Discussion

The result of this meta-analysis undoubtedly showed the beneficial effects of acupuncture in the management of side effects from drug therapies on patients with breast cancer. Symptoms, including gastrointestinal disorders, neuropathy, arthralgia, and congestive impairment, can affect patients, from daily life behaviours to self-care ability. It is promising and encouraging for patients with breast cancer to have a clinically significant adjuvant therapy that can alleviate the side effects of drug therapy. Their quality of life during and after the treatment period could be dramatically enhanced.

The meta-analysis results were consistent and robust in the overall primary outcome, as well as in the subgroup analysis. The purpose of the subgroup analysis was to ensure that acupuncture is effective in reducing the common side effects. The results of all the analysis proved that acupuncture could clinically reduce all four symptoms induced by drug therapy. As there are more than one measurement and index in the same group of analysis, the heterogeneity was elevated in some of the studies. As a response, the random-effect model was chosen, and sensitivity analysis was carried out to assess the effect of high heterogeneity on the results of the forest plot [28]. It was confirmed that although the calculated heterogeneity was higher than ideal, the results were statistically significant, especially with those minimal *p* values. The unanimous results in some of the subgroup analyses were strong evidence suggesting the advantageous effects of acupuncture as adjuvant therapy. On top of that, the sensitivity analysis also confirmed that the results were consistent in nearly all the subgroups. Excluding the trial results with high heterogeneity in a coherent beneficial effect, only the AIAA subgroup resulted in a nonsignificant effect. We are confident that even if the heterogeneity of the meta-analysis was higher than ideal, the significance of the calculated effects would not diminish.

A characteristic of this study is that the duration of treatments, as well as the treatment scheme, was not the same in every RCT. Acupuncture is a kind of traditional Chinese medicinal therapy that involves the stimulation of various acupoints using invasive needles or noninvasive methods. It follows the same philosophy as with Chinese medicine, which states that the complex treatment is tailored and finely tuned according to each patient's body conditions and disease situation [29]. It is almost impossible to have the same formulated acupuncture “prescription” for every patient. Alternatively, on the other hand, the identical acupuncture treatment would have a different therapeutic response on every patient [30]. It is impossible to recruit enough RCTs and sample size with the same treatment scheme in a meta-analysis in this field of study. The best possible information on the treatment regimens is to find out the most commonly used acupoints to treat a symptom. In general, a majority choice of acupoints in the treatment involved the conception vessel (*Renmai*), namely, CV4 *Guanyuan*, CV6 *Qihai*, and CV12 *Zhongwan*.

Due to the nature of acupuncture treatment, it is challenging to design and perform a sham control treatment. Recent studies usually involve the usage of retractable needles, superficial invasion in nonacupoints, or nonelectric applied needles as a sham control [31]. However, studies are suggesting that sham acupuncture may be associated with physiological effects by causing the release of endorphins and pain-killing substances [32, 33]. Therefore, we could not fully remove the impact of the placebo effects in acupuncture clinical trials, even with or without sham control or waitlist control group [34, 35]. Nevertheless, in the present study, patients were having a significantly greater benefit on the real acupuncture arms than the control arm RCTs with sham control or no-treatment controls.

**Figure 7:**
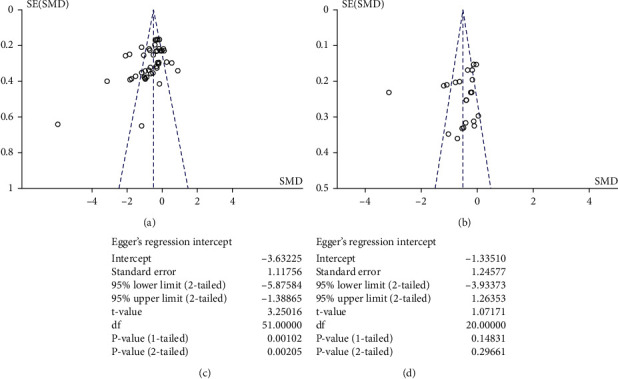
Funnel plots of (a) primary outcome and (b) secondary outcome of all included studies. ((c) and (d)) Egger's test statistics on the risk of bias assessment on the primary and the secondary outcomes.

Another limitation of this study is the limiting number of RCTs and patients included. With our search strategies and criteria, we allow only RCTs with invading needles included, but not acupressure, moxibustion, massage, and so on, in order to narrow down and illustrate the accurate effects of real acupuncture. However, this limits the ability of this study to discover the potential effects of other forms of alternative treatments. The acceptance of invading acupuncture may not be as wide as the other methods. Patients agreeing to join the clinical trials tend to accept and believe in the effect of acupuncture beforehand, and a certain level bias may occur in the patient selection [36]. The numbers and reasons for drop-out and withdrawals were not reported in some of the studies, which may lead to potential reporting bias. Only studies published in English and Chinese were included, as studies in other languages cannot be assessed. A limitation in the patients' origins and races could result in selection bias. Moreover, in general, clinical trials with positive results were more commonly published as research articles, while trials with incomplete or negative results would be less reported [37, 38]. The opposite effects reported by Bao et al. in 2013 provided an insight into this possibility [16]. In this study, not all the registered clinical trials without published results were considered and included. The risk of publication bias across studies was assessed by a funnel plot (Figure 7(a)). There were a considerable number of spots lying outside of the funnel region. A slight but not major bias was observed towards the real acupuncture side (negative). Egger's test statistics suggested that there was a small bias leaning towards the negative side (intercept = −3.63, *t*-value = 3.25, *p* value = 0.001) (Figure 7(c)). Due to the nature of clinical trials as previously discussed, the remarkable effect observed could justify this small publication bias. For the secondary outcome, all studies were lying within or very close to the funnel area. Only one outlier was observed, and no risk of publication bias was suggested in the study (Figure 7(b)). Egger's test statistics showed no sign of publication bias (intercept = −0.134, *t*-value = 1.07, *p* value = 0.148) (Figure 7(d)).

The safety of using acupuncture as adjuvant therapy in cancer treatment has been a great concern. In our study, there were no severe adverse events reported in any of the studies. Most of the unpleasant feelings due to the acupuncture treatment were reported during the treatment. Only mild side effects such as bruising and faintness were found in a few cases after or in between treatment visits (Table 3). We strongly suggest the safety of acupuncture treatments according to this study. Acupuncture performed by experienced acupuncturists has been proved to be extraordinarily safe and harmless [39, 40]. Therefore, acupuncture is definitely worth recommended for patients with breast cancer receiving drug therapy. Acupuncture can manage the side effects of drug therapies with minimal risk and promising benefits.

After reviewing hundreds of acupuncture-related clinical trials, there are several suggestions on future study practice. First of all, a more standardised treatment scheme should be considered in RCTs to increase the creditability and quality control of the trials. Duration, treatment cycles, acupoints, and electrofrequencies are parameters that should be regulated. In addition, a more promising control design should be developed in RCTs involving acupuncture treatment. Further large-scale clinical studies on the effect of sham control could be performed to evaluate the optimal design. Regarding acupuncture in patients receiving drug therapies, more objective measurement indices could be considered, as many of the scales used currently were from patient-reported measures.

## 5. Conclusion

This meta-analysis showed that acupuncture could effectively reduce the side effects induced by drug therapies in patients with breast cancer. The symptoms with positive responses are gastrointestinal disorders, chemotherapy-induced peripheral neuropathy, aromatase inhibitor-associated arthralgia, aromatase inhibitor-associated joint symptoms, and cognitive impairment. Moreover, the quality of life of patients has enhanced to a certain extent. Without severe adverse events reported, we recommend that suitable acupuncture treatments are considered as an excellent adjuvant therapy with drug therapies in patients with breast cancer. Future RCTs with standardised treatment scheme and longer terms of follow-up are needed to further evaluate the roles and benefits of acupuncture as adjuvant therapies in different diseases.

## Figures and Tables

**Table 1:** Characteristics of the included studies.

Study ID	Sample size (*T*/*C*)	Design	Baseline characteristics	Intervention group	Control group	Duration	Primary outcome measures	Secondary outcome measures
Bao et al. 2013 [16]	47 (23/24)	Dual-centre double-blind RCT	Age range: 44–82 Median duration of therapy: 426 days Disease stage: N/A	CV4, CV6, CV12, bilateral LI4, MH6, GB34, ST36, KI3, BL65	Sham acupuncture (nonpenetrating retractable needles)	20 min, 8 weeks, weekly	(1) HAQ-DI (2) pain VAS scores	N/A
Crew et al. 2010 [15]	38 (20/18)	Single-centre double-blind RCT	Age range: 37–77 Median duration of therapy: 7/12 months Disease stage: I, II, III	Standard TCM point prescription (not given)	Sham acupuncture (superficial needle insertion at non-acupoints)	30 min, 6 weeks, biweekly	(1) BPI (2) WOMAC (3) M-SACRAH	FACT-G
Greenlee et al. 2016 [18]	48 (25/23)	Single-centre double-blind RCT	Age range: 27–79 Median duration of therapy: 12 cycles Disease stage: I, II, III	GB34, ST37, LI4, LI10, L3, L5, C5, C7	Sham acupuncture (park sham collapsible needles at nonacupoints)	30 min, 12 weeks, weekly	(1) BPI-SF (2) FACT-NTX (3) NPS-4	FACT-TAX
He 2017 [13]	64 (32/32)	Single-centre RCT	Age range: 18–75 Disease stage: I, II, IIIA	CV12, bilateral LV13, CV6, ST25, PC6, ST36	Sham acupuncture (superficial needle insertion at nonacupoints)	30 min, 1 week, thrice per week	(1) nausea level (0–3)	HADS
Hershman et al. 2018 [21]	155 (101/54)	Multi-centre double-blind RCT	Age range: 34.1–80.6 Median duration of therapy: 1 year Disease stage: I, II, III	Standards for reporting of controlled trials in acupuncture	Sham acupuncture (superficial needle insertion at nonacupoints)	30–45 min, 1–6 weeks biweekly, 7–12 weeks weekly	(1) BPI-WP (2) BPI-SF (3) WOMAC (4) M-SACRAH	(1) FACT-ES (2) PROMIS PI-SF
Li 2017 [20]	40 (20/20)	Single-centre RCT	Mean age (SD): 46.1 (9.369) KPS: >80 Disease stage: I, II, III, IV	CV6, CV4, bilateral ST36, SP6, ST25, LV3, PC6	No control treatment	30 min, once	(1) Nausea level (0–3) (2) Vomiting level (0–3)	N/A
Liu 2014 [17]	140 (70/70)	Single-centre RCT	Mean age (SD): 51.16 (10.25) Mean KPS (SD): 95.26 (1.38)	CV12, CV10, CV6, CV4, bilateral ST25, SP15, ST24	No control treatment	30 min, 3 days, twice everyday	(1) Nausea level (0–3) (2) Vomiting level (0–3) (3) Appetite level (0–3) (4) Constipation score (0–5) (5) Diarrhea score (0–5)	N/A
Lu et al. 2020 [26]	33 (16/17)	Single-centre waitlist RCT	Age range: 26–71 Median duration of therapy: 17.3/13.3 Disease stage: I, II, III	Yin Tang, LI11, TW5, Baxie, SP9, ST36, SP6, K3, LR3, Qiduan	Waitlist control	30 min, 8 weeks, 18 treatment	(1) PNQ (2) FACT-NTX (3) BPI-SF	EORTC QLQ-C30
Mao et al. 2014 [12]	41 (21/20)	Single-centre RCT	Joint pain for at least 3mo Disease stage: I, II, III BPI-WP: ≥4	4 local acupoints around the pain joint (not provided)	Sham acupuncture (nonpenetrating needles at nonacupoints)	30 min, biweekly for 2 weeks, weekly for next 6 weeks	(1) BPI	HADS
Quinlan-Woodward et al. 2016 [19]	30 (15/15)	Single-centre blind RCT	Mean age (SD): 58.1 (11.5) All Caucasians	Standard TCM point prescription (not given)	No control treatment	36 min, once	(1) Nausea level (0–10)	N/A
Shen et al. 2000 [14]	70 (37/33)	Single-centre double-blind RCT	Mean age (SD): 44.7 (8.0) KPS: >80	PC6, ST36	Sham acupuncture (superficial needle insertion near LU7, GB34)	20 min, 5 days, once every day	(1) Emesis episodes	N/A
Shi 2019 [24]	169 (85/84)	Single-centre RCT	Age range: 18–75 KPS: >60	GV20, GV4, GV3, GV2, CV12, CV4, CV6, CV3,	Sham acupuncture (superficial needle insertion 20 mm next to real acupoints)	30 min, 7 days, once every day	(1) Nausea level (0–4) (2) Vomiting level (0–4)	(1) SAS (2) SDS (3) KPS
Tong et al. 2018 [22]	75 (39/36)	Single-centre blind RCT	Age range: 21–55 (premenopausal) Disease stage: I, II	DU20, EX-HN1, KI3, DU24, KI4, GB39, ST36	No control treatment	30 min, 5 days per week, 4 weeks for 2 courses	(1) FACT-COG (2) AVLT	FACT-COG
Zhang 2018 [23]	93 (48/45)	Single-centre RCT	Age range: 25–55 MoCA: <26 MMSE: 21–26	DU20, EM1, D24, PC6, HT7, GB20, CV17, CV12, CV6	Sham acupuncture (superficial needle insertion at nonacupoints)	30 min, 8 weeks, biweekly	(1) MoCA (2) MMSE	N/A
Zhang 2019 [25]	104 (53/51)	Single-centre RCT	Age range: 30–55 Disease stage: I, II, III, IV Patients with cognitive impairment	SP10, CV17, CV12, CV6, DU20, DU16, bilateral ST36, BI15, BI45, HT5, KD6	Sham acupuncture (superficial needle insertion at nonacupoints)	20 min, 8 weeks, biweekly	(1) MoCA (2) MMSE	EORTC QLQ-C30
Zhang et al. 2020 [27]	83 (40/43)	Single-centre double-blind RCT	Mean age (SD): 47.9 (10.6) Disease stage: I, II, IIIA Under chemo (%): 73.3/78.7	HT7, LI4, TE5, ST36, ST40, CV12, CV4, GV26, bilateral SP6	Minimum acupuncture stimulation, bilateral BL7, LI10, BL59	30 min, 8 weeks, biweekly	(1) MoCA (2) Forward digit span test (3) Reverse digit span test	N/A

Abbreviations are listed in Table S1.

**Table 2:** Assessment of certainty of evidence using the GRADE approach.

Certainty assessment						Certainty	
Name of study	Risk of bias	Indirectness	Inconsistency	Imprecision	Publication bias		

Bao et al. 2013 [16]	Not serious	Not serious	Serious	Serious	Low probability	⊕⊕○○	Low
Crew et al. 2010 [15]	Not serious	Not serious	Serious	Not serious	High probability	⊕⊕○○	Low
Greenlee et al. 2016 [18]	Serious	Not serious	Not serious	Not serious	Low probability	⊕⊕⊕○	Moderate
He 2017 [13]	Serious	Not serious	Not serious	Not serious	Low probability	⊕⊕⊕○	Moderate
Hershman et al. 2018 [21]	Not serious	Not serious	Not serious	Not serious	Low probability	⊕⊕⊕⊕	High
Li 2017 [20]	Serious	Not serious	Not serious	Not serious	Low probability	⊕⊕⊕○	Moderate
Liu 2014 [17]	Serious	Not serious	Not serious	Not serious	High probability	⊕⊕○○	Low
Lu et al. 2020 [26]	Serious	Not serious	Not serious	Not serious	Low probability	⊕⊕⊕○	Moderate
Mao et al. 2014 [12]	Not serious	Not serious	Not serious	Not serious	Low probability	⊕⊕⊕⊕	High
Quinlan-Woodward et al. 2016 [19]	Serious	Not serious	Not serious	Not serious	Low probability	⊕⊕⊕○	Moderate
Shen et al. 2000 [14]	Not serious	Not serious	Not serious	Not serious	High probability	⊕⊕⊕○	Moderate
Shi 2019 [24]	Not serious	Not serious	Serious	Serious	High probability	⊕○○○	Very low
Tong et al. 2018 [22]	Serious	Not serious	Not serious	Not serious	Low probability	⊕⊕⊕○	Moderate
Zhang 2018 [23]	Not serious	Not serious	Serious	Not serious	High probability	⊕⊕○○	Low
Zhang 2019 [25]	Not serious	Not serious	Serious	Not serious	High probability	⊕⊕○○	Low
Zhang et al. 2020 [27]	Not serious	Not serious	Not serious	Not serious	Low probability	⊕⊕⊕⊕	High

**Table 3:** Significant side effects or adverse events reported in the trials.

Study ID	Intervention group	Side effects or adverse events
Bao et al. 2013 [16]	CV4, CV6, CV12, bilateral LI4, MH6, GB34, ST36, KI3, BL65	No significant adverse events were reported in both arms.
Crew et al. 2010 [15]	Standard TCM point prescription (not given)	Three out of 38 patients felt moderately painful during the acupuncture treatment. No other adverse events were reported.
Greenlee et al. 2016 [18]	GB34, ST37, LI4, LI10, L3, L5, C5, C7	One patient showed adverse event with discomfort, minor swelling, and bruising on an acupuncture site after needle withdrawal.
He 2017 [13]	CV12, bilateral LV13, CV6, ST25, PC6, ST36	No significant adverse events were reported in both arms.
Hershman et al. 2018 [21]	Standards for reporting of controlled trials in acupuncture	47% and 25% patients were experiencing grade 1 bruising in the true and sham acupuncture group. One patient from each group showed grade 2 presyncope once.
Li 2017 [20]	CV6, CV4, bilateral ST36, SP6, ST25, LV3, PC6	80% of the patients felt no to mild pain during the acupuncture. No significant adverse events were reported in both arms.
Liu 2014 [17]	CV12, CV10, CV6, CV4, bilateral ST25, SP15, ST24	One out of 140 patients showed presyncope after acupuncture and recovered after treatment. No other significant adverse events were reported.
Lu et al. 2020 [26]	Yin tang, LI11, TW5, Baxie, SP9, ST36, SP6, K3, LR3, Qiduan	No significant adverse events were reported in both arms. One patient-reported grade 1 pruritus in the feet and one reported grade 2 joint pain.
Mao et al. 2014 [12]	4 local acupoints around the pain joint (not provided)	No significant adverse events were reported in both arms.
Quinlan-Woodward et al. 2016 [19]	Standard TCM point prescription (not given)	No significant adverse events were reported in both arms.
Shen et al. 2000 [14]	PC6, ST36	One patient experienced shock sensation from needle stimulator apparatus once. Another patient had aggravated tingling sensation on residual peripheral neuropathy following each needling procedure. No other significant adverse events were reported.
Shi 2019 [24]	GV20, GV4, GV3, GV2, CV12, CV4, CV6, CV3,	No significant adverse events were reported in both arms.
Tong et al. 2018 [22]	DU20, EX-HN1, KI3, DU24, KI4, GB39, ST36	No significant adverse events were reported in both arms.
Zhang 2018 [23]	DU20, EM1, D24, PC6, HT7, GB20, CV17, CV12, CV6	No significant adverse events were reported in both arms.
Zhang 2019 [25]	SP10, CV17, CV12, CV6, DU20, DU16, bilateral ST36, BI15, BI45, HT5, KD6	No significant adverse events were reported in both arms.
Zhang et al. 2020 [27]	HT7, LI4, TE5, ST36, ST40, CV12, CV4, GV26, bilateral SP6	No significant adverse events were reported in both arms.

## Data Availability

The data used to support the findings of this study are included within the article and the supplementary information files.

## Supplementary Materials

Supplementary Materials. S1: list of abbreviations. S2: list of detailed search syntax for each database.

## References

[1] Ferlay J., Soerjomataram I., Ervik M. (2013). *GLOBOCAN 2012 Cancer Incidence and Mortality Worldwide: IARC Cancerbase No. 11*.

[2] Gradishar W. J., Anderson B. O., Balassanian R. (2017). NCCN guidelines insights: breast cancer, version 1.2017. *Journal of the National Comprehensive Cancer Network: Journal of the National Comprehensive Cancer Network*.

[3] Shapiro C. L., Recht A. (2001). Side effects of adjuvant treatment of breast cancer. *New England Journal of Medicine*.

[4] Tao J. J., Visvanathan K., Wolff A. C. (2015). Long term side effects of adjuvant chemotherapy in patients with early breast cancer. *The Breast*.

[5] VanderPloeg K., Yi X. (2009). Acupuncture in modern society. *Journal of Acupuncture and Meridian Studies*.

[6] Pan Y., Yang K., Shi X. (2018). Clinical benefits of acupuncture for the reduction of hormone therapy-related side effects in breast cancer patients: a systematic review. *Integrative Cancer Therapies*.

[7] Roberts K., Rickett K., Greer R., Woodward N. (2017). Management of aromatase inhibitor induced musculoskeletal symptoms in postmenopausal early breast cancer: a systematic review and meta-analysis. *Critical Reviews in Oncology*.

[8] Kim W., Lee W.-B., Lee J.-W. (2015). Traditional herbal medicine as adjunctive therapy for breast cancer: a systematic review. *Complementary Therapies in Medicine*.

[9] Moher D., Liberati A, Tetzlaff J, Altman D. G (2009). Preferred reporting items for systematic reviews and meta-analyses: the PRISMA statement. *PLoS Medicine*.

[10] Higgins J. P., Green S. (2019). *Cochrane Handbook for Systematic Reviews of Interventions*.

[11] Higgins J., Deeks J. (2011). Obtaining standard deviations from standard errors and confidence intervals for group means. *Cochrane Handbook for Systematic Reviews of Interventions*.

[12] Mao J. J., Farrar J. T., Bruner D. (2014). Electroacupuncture for fatigue, sleep, and psychological distress in breast cancer patients with aromatase inhibitor‐related arthralgia: a randomized trial. *Cancer*.

[13] He P. S., Pan G. F., Wang X. M. (2017). Prospective randomized controlled trial of treating and preventing chemotherapy-related nausea and vomiting on breast cancer patients with “experienced ten acupoints”. *China Journal of Traditional Chinese Medicine and Pharmacy*.

[14] Shen J., Wenger N., Glaspy J. (2000). Electroacupuncture for control of myeloablative chemotherapy-induced emesis. *JAMA*.

[15] Crew K. D., Capodice J. L., Greenlee H. (2010). Randomized, blinded, sham-controlled trial of acupuncture for the management of aromatase inhibitor-associated joint symptoms in women with early-stage breast cancer. *Journal of Clinical Oncology*.

[16] Bao T., Cai L., Giles J. T. (2013). A dual-center randomized controlled double blind trial assessing the effect of acupuncture in reducing musculoskeletal symptoms in breast cancer patients taking aromatase inhibitors. *Breast Cancer Research and Treatment*.

[17] Liu D. (2014). *Clinical Research of Acute Gastrointestinal Side Effects of Chemotherapy in Breast Cancer Treated by Bo’s Abdominal Acupuncture*.

[18] Greenlee H., Crew K. D., Capodice J. (2016). Randomized sham-controlled pilot trial of weekly electro-acupuncture for the prevention of taxane-induced peripheral neuropathy in women with early stage breast cancer. *Breast Cancer Research and Treatment*.

[19] Quinlan-Woodward J., Gode A., Dusek J., Reinstein A., Johnson J., Sendelbach S. (2016). Assessing the impact of acupuncture on pain, nausea, anxiety, and coping in women undergoing a mastectomy. *Oncology Nursing Forum*.

[20] Li D. (2017). *Effect of Acupuncture Combined 5-HT Receptor Antagonist on Acute Gastrointestinal Reaction after Breast Cancer Chemotherapy*.

[21] Hershman D. L., Unger J. M., Greenlee H. (2018). Effect of acupuncture vs sham acupuncture or waitlist control on joint pain related to aromatase inhibitors among women with early-stage breast cancer. *JAMA*.

[22] Tong T., Pei C., Chen J., Lv Q., Zhang F., Cheng Z. (2018). Efficacy of acupuncture therapy for chemotherapy-related cognitive impairment in breast cancer patients. *Medical Science Monitor*.

[23] Zhang C., Han D., Zhang Y., Li N., Zhang Q. (2018). Clinical study on acupuncture treatment of Yiqi-Tiaoshen acupoint on mild cognitive impairment caused by chemotherapy of breast cancer patients. *Guiding Journal of Traditional Chinese Medicine and Pharmacy*.

[24] Shi X. (2019). *Clinical Study on the Side-Effects of “TiaoRen Tongdu” Acupuncture Method on Chemotherapeutic Drugs for Breast Cancer*.

[25] Zhang Y., Zhang C., Xu X. H., Zhang Q. (2019). Clinical observation on acupuncture of reconciling qi and blood, tonifying heart and mind method in the treatment of 53 patients of breast cancer chemotherapy-related cognitive impairment with disorder of qi and blood syndrome. *Journal of Traditional Chinese Medicine*.

[26] Lu W., Giobbie‐Hurder A., Freedman R. A. (2020). Acupuncture for chemotherapy‐induced peripheral neuropathy in breast cancer survivors: a randomized controlled pilot trial. *The Oncologist*.

[27] Zhang Z. J., Man S.-C., Yam L.-L. (2020). Electroacupuncture trigeminal nerve stimulation plus body acupuncture for chemotherapy-induced cognitive impairment in breast cancer patients: an assessor-participant blinded, randomized controlled trial. *Brain, Behavior, and Immunity*.

[28] Bown M. J., Sutton A. J. (2010). Quality control in systematic reviews and meta-analyses. *European Journal of Vascular and Endovascular Surgery*.

[29] Kaptchuk T. J. (2002). Acupuncture: theory, efficacy, and practice. *Annals of Internal Medicine*.

[30] Chon T. Y., Lee M. C. (2013). Acupuncture. *Mayo Clinic Proceedings*.

[31] Park J., White A., Stevinson C., Ernst E., James M. (2002). Validating a new non-penetrating sham acupuncture device: two randomised controlled trials. *Acupuncture in Medicine*.

[32] Lund I., Lundeberg T. (2006). Are minimal, superficial or sham acupuncture procedures acceptable as inert placebo controls?. *Acupuncture in Medicine*.

[33] Zhang L.-L., Chu Q., Wang S., Lai H., Xie B.-B. (2016). Is sham acupuncture as effective as traditional Chinese acupuncture? It’s too early to say. *Chinese Journal of Integrative Medicine*.

[34] Kong J., Spaeth R., Cook A. (2013). Are all placebo effects equal? Placebo pills, sham acupuncture, cue conditioning and their association. *PLoS One*.

[35] Appleyard I., Lundeberg T., Robinson N. (2014). Should systematic reviews assess the risk of bias from sham-placebo acupuncture control procedures?. *European Journal of Integrative Medicine*.

[36] Han X.-Y., Li X., Liang N. (2019). Factors influencing the quality of clinical trials on traditional Chinese medicine-qualitative interviews with trial auditors, clinicians and academic researchers. *Complementary Therapies in Clinical Practice*.

[37] Ioannidis J. P., Caplan A. L., Dal-Ré R. (2017). Outcome reporting bias in clinical trials: why monitoring matters. *BMJ*.

[38] Su C.-X., Han M., Ren J. (2015). Empirical evidence for outcome reporting bias in randomized clinical trials of acupuncture: comparison of registered records and subsequent publications. *Trials*.

[39] Deng G., Bao T., Mao J. J. (2018). Understanding the benefits of acupuncture treatment for cancer pain management. *Oncology*.

[40] Birch S., Lee M. S., Alraek T., Kim T.-H. (2019). Evidence, safety and recommendations for when to use acupuncture for treating cancer related symptoms: a narrative review. *Integrative Medicine Research*.

