# Supplementary material for: Systematic Review with Meta-Analysis: Effectiveness and Safety of Acupuncture as Adjuvant Therapy for Side Effects Management in Drug Therapy-Receiving Breast Cancer Patients
Source: Evid Based Complement Alternat Med. 2021 Oct 12;2021:9949777. doi: 10.1155/2021/9949777 (PMC8526206; doi:10.1155/2021/9949777)
Supplement: Supplementary Materials. — S1: list of abbreviations. S2: list of detailed search syntax for each database. [file 9949777.f1.docx]

**Table S1.** List of Abbreviations.

| AIAA | Aromatase Inhibitor-Associated Arthralgia |
| --- | --- |
| AIAS | Aromatase Inhibitor-Associated joint problems |
| AMED | Allied and complementary Medicine Database |
| AVLT | Auditory-Verbal Learning Test |
| BPI | Brief Pain Inventory |
| BPI-SF | Brief Pain Inventory-Short Form |
| BPI-WP | Brief Pain Inventory-Worst Pain |
| CI | Confidence Interval |
| CINAHL Plus | Cumulative Index to Nursing and Allied Health Literature Plus |
| CIPN | Chemotherapy-Induced Peripheral Neuropathy |
| CNKI | China National Knowledge Infrastructure |
| EMBASE | Excerpta Medica dataBASE |
| EORTC QLQ-C30 | European Organisation for Research and Treatment of Cancer Quality-of-Life Questionnaire Core-30 |
| FACT-COG | The Functional Assessment of Cancer Treatment-Cognition |
| FACT-ES | The Functional Assessment of Cancer Therapy-Endocrine Symptoms |
| FACT-G | The Functional Assessment of Cancer Therapy-General |
| FACT-NTX | The Functional Assessment of Cancer Therapy-Neurotoxicity |
| FACT-TAX | The Functional Assessment of Cancer Therapy-Taxane |
| HADS | Hospital Anxiety and Depression Scale |
| HAQ-DI | Health Assessment Questionnaire Disability Index |
| KPS | Karnofsky Performance Status scale |
| MEDLINE | Medical Literature Analysis and Retrieval System Online |
| MMSE | Mini-Mental State Examination |
| MoCA | Montreal Cognitive Assessment |
| M-SACRAH | Modified Score for the Assessment of Chronic Rheumatoid Affections of the Hands |
| NPS | Neuropathic Pain Scale |
| PNQ | Patient Neurotoxicity Questionnaire |
| PROMISPI-SF | Patient-Reported Outcomes Measurement Information System Pain Impact-Short Form |
| QoL | Quality of Life |
| RCTs | Ramdomized Controlled Trials |
| SAS | Self-rating Anxiety Scale |
| SD | Standard Deviation |
| SDS | Self-rating Depression Scale |
| SMD | Standard Mean Difference |
| VAS | Visual Analog Scale |
| WOMAC | Western Ontario and McMaster Universities Osteoarthritis Index |

**Table S2.** Database search syntax

Cochrane Library

| **ID** | **Search syntax** |
| --- | --- |
| #1 | chemotherapy |
| #2 | 'therapy, drug' or 'drug therap*' or 'therapies, drug' or 'chemotherap*' or 'pharmacotherap*' or 'pharmacologic therap*' or chemotherapies or vincristine or oncovin or vinblastine or vinorelbine or vindesine or vinorelbine or vinca alkaloid or vinca alkaloids or oxaliplatin or eloxatin or cisplatin or carboplatin or platinum or platinums or taxane or taxanes or docetaxel or paclitaxel or ifosfamide or ifex or Ixabepilone or epothilone or Epothilones or Bortezomib or proteasome inhibitor or proteasome inhibitors or Thalidomide or Lenalidomide or procarbazine or thiotepa or podophyllin or topoisomerase inhibitor or teniposide or etoposide or vepesid or vumon or gemcitabine or Induction Chemotherapy or Chemotherapy, Adjuvant or Consolidation Chemotherapy or Maintenance Chemotherapy |
| #3 | nausea or vomiting |
| #4 | nausea or antiemetic* or emetic* or emesis or vomiting |
| #5 | ('chemotherapy induced nausea' and vomiting) or ('chemotherapy-induced nausea' and vomiting) or cinv |
| #6 | arthralgia or 'aromatase inhibitor-associated arthralgia' |
| #7 | (aromatase NEAR/3 (inhibit* or anastrozole or exemestane or letrozole or aminoglutethimide* or atamestane or formestane or vorozole or arimidex or aromasin or femara or fadrozole or lentaron or rivizor or cytadren)) |
| #8 | ('aromatase inhibitor-associated' NEAR/3 (arthralgia or musculoskelet*)) or ('aromatase inhibitor associated' NEAR/2 (arthralgia or musculoskelet*)) or 'aia' or 'ai' |
| #9 | neurotoxicit* |
| #10 | 'peripher* NEAR/3 (neuropathy or neuropath* or neurotoxicit*) or cipn |
| #11 | ('chemotherapy-induced peripheral' and neurotoxicit*) or ('chemotherapy induced peripheral' and neurotoxicit*) or ('chemotherapy-induced peripheral' and neuropath*) or ('chemotherapy induced peripheral' and neuropath*) |
| #12 | chemobrain* |
| #13 | cognitive function impairment |
| #14 | ('cognitive impair*' or 'cogniti*' or 'neuropsycholog*' or 'acute brain syndrome' or 'acute cerebral insufficiency' or 'acute confusion' or 'acute confusional state' or 'cognitive defect' or (cognitive NEAR/2 (dysfunction or decline)) or (mental NEAR/2 deterioration) or 'brain disease' or 'brain disorders') |
| #15 | #1 or #2 |
| #16 | #3 or #4 or #5 |
| #17 | #6 or #7 or #8 |
| #18 | #9 or #10 or #11 |
| #19 | #12 or #13 or #14 |
| #20 | breast neoplasm |
| #21 | (breast or mamma*) NEAR/3 (cancer* or neopla* or adenocarcin* or carcin* or tumor* or tumour* or malignan* or sarcoma* or mass* or DCIS or ductal* or infiltrat* or intraductal* or lobula* or medullary) |
| #22 | acupuncture |
| #23 | acupunctur* or acupoint* or acupuncture therap* or electroacupunctur* or electroacupuncture therap* or "zhen jiu" or "zhenjiu" or 'dry needle' |
| #24 | #20 or #21 |
| #25 | #22 or #23 |
| #26 | #15 and #24 and #25 |
| #27 | (#16 or #17 or #18 or #19) and #26 |
| #28 | double-blind procedure or "randomized controlled trial" or "single-blind procedure" or random* or factorial* or placebo* or "doubl* blind*" or "singl* blind*" or assign* or allocat* or volunteer* |
| #29 | #27 and #28 |

Web of Science

| **ID** | **Search syntax** |
| --- | --- |
| #1 | TS=chemotherapy |
| #2 | TS=('therapy, drug'  or  'drug  therap*'  or  'therapies,  drug'  or  'chemotherap*'  or  'pharmacotherap*'  or  'pharmacologic  therap*'  or  chemotherapies  or  vincristine  or  oncovin  or  vinblastine  or  vinorelbine  or  vindesine  or  vinorelbine  or  'vinca  alkaloid'  or  'vinca  alkaloids'  or  oxaliplatin  or  eloxatin  or  cisplatin  or  carboplatin  or  platinum  or  platinums  or  taxane  or  taxanes  or  docetaxel  or  paclitaxel  or  ifosfamide  or  ifex  or  Ixabepilone  or  epothilone  or  Epothilones  or  Bortezomib  or  'proteasome  inhibitor'  or  'proteasome  inhibitors'  or  Thalidomide  or  Lenalidomide  or  procarbazine  or  thiotepa  or  podophyllin  or  'topoisomerase  inhibitor'  or  teniposide  or  etoposide  or  vepesid  or  vumon  or  gemcitabine  or  'Induction  Chemotherapy'  or  'Chemotherapy,  Adjuvant'  or  'Consolidation  Chemotherapy'  or  'Maintenance  Chemotherapy') |
| #3 | TS=(nausea or  vomiting) |
| #4 | TS=(nausea or  'antiemetic*'  or  'emetic*'  or  emesis  or  vomiting) |
| #5 | TS=(('chemotherapy induced  nausea'  and  'vomiting')  or  ('chemotherapy-induced nausea' and 'vomiting')  or  cinv) |
| #6 | TS=(arthralgia or  'aromatase  inhibitor-associated  arthralgia') |
| #7 | TS=(aromatase NEAR  (inhibit* or anastrozole or exemestane or letrozole or aminoglutethimide* or atamestane or formestane or vorozole or arimidex or aromasin or femara or fadrozole or lentaron or rivizor or cytadren) ) |
| #8 | TS=(('aromatase inhibitor-associated'  NEAR  ('arthralgia' or 'musculoskelet*') )  or  ('aromatase inhibitor associated NEAR ('arthralgia' or 'musculoskelet*') )  or  'aia'  or  'ai') |
| #9 | TS=(neurotoxicity) |
| #10 | TS=('peripher*' NEAR  ('neuropathy' or 'neuropath*' or 'neurotoxicit*')  or  'cipn') |
| #11 | TS=((('chemotherapy-induced peripheral'  and  'neurotoxicit*')  or  ('chemotherapy induced peripheral' and 'neurotoxicit*')  or  ('chemotherapy-induced peripheral' and 'neuropath*')  or  ('chemotherapy induced peripheral' and 'neuropath*') )) |
| #12 | TS=(chemobrain*) |
| #13 | TS=(‘cognitive function  impairment’) |
| #14 | TS=('cognitive impair*'  or  'cogniti*'  or  'neuropsycholog*'  or  'acute  brain  syndrome'  or  'acute  cerebral  insufficiency'  or  'acute  confusion'  or  'acute  confusional  state'  or  'cognitive  defect'  or  'cognitive  dysfunction'  or  'cognitive  decline'  or  'mental  deterioration'  or  'brain  disease'  or  'brain  disorders') |
| #15 | #1 OR #2 |
| #16 | #3 OR #4 OR #5 |
| #17 | #6 OR #7 OR #8 |
| #18 | #9 OR #10 OR #11 |
| #19 | #12 OR #13 OR #14 |
| #20 | TS=(breast neoplasm) |
| #21 | TS=((breast or  mamma*)  NEAR  (cancer* or neopla* or adenocarcin* or carcin* or tumor* or tumour* or malignan* or sarcoma* or mass* or DCIS or ductal* or infiltrat* or intraductal* or lobula* or medullary) ) |
| #22 | TS=(acupuncture) |
| #23 | TS=(acupunctur* or  acupoint*  or  acupuncture  therap*  or  electroacupunctur*  or  electroacupuncture  therap*  or  "zhen  jiu"  or  "zhenjiu"  or  'dry  needle') |
| #24 | #20 OR #21 |
| #25 | #22 OR #23 |
| #26 | #15 AND #24 AND #25 |
| #27 | (#16 OR #17 OR #18 OR #19) AND #26 |
| #28 | TS=("double-blind procedure"  or  "randomized  controlled  trial"  or  "single-blind  procedure"  or  random*  or  factorial*  or  placebo*  or  "doubl*  blind*"  or  "singl*  blind*"  or  assign*  or  allocat*  or  volunteer*) |
| #29 | #27 AND #28 |

Pubmed

| **ID** | **Search syntax** |
| --- | --- |
| #1 | drug therapy or 'therapy, drug' or drug therap* or 'therapies, drug' or chemotherap* or pharmacotherap* or pharmacologic therap* or chemotherapies or vincristine or oncovin or vinblastine or vinorelbine or vindesine or vinorelbine or 'vinca alkaloid' or 'vinca alkaloids' or oxaliplatin or eloxatin or cisplatin or carboplatin or platinum or platinums or taxane or taxanes or docetaxel or paclitaxel or ifosfamide or ifex or Ixabepilone or epothilone or Epothilones or Bortezomib or 'proteasome inhibitor' or 'proteasome inhibitors' or Thalidomide or Lenalidomide or procarbazine or thiotepa or podophyllin or 'topoisomerase inhibitor' or teniposide or etoposide or vepesid or vumon or gemcitabine or 'Induction Chemotherapy' or 'Chemotherapy, Adjuvant' or 'Consolidation Chemotherapy' or 'Maintenance Chemotherapy' |
| #2 | nausea or vomiting or antiemetic* or emetic* or emesis or ('chemotherapy induced nausea' and 'vomiting') or ('chemotherapy-induced nausea' and 'vomiting') or cinv |
| #3 | arthralgia or 'aromatase inhibitor-associated arthralgia' or (aromatase and (inhibit* or anastrozole or exemestane or letrozole or aminoglutethimide* or atamestane or formestane or vorozole or arimidex or aromasin or femara or fadrozole or lentaron or rivizor or cytadren)) or ('aromatase inhibitor-associated' and (arthralgia or musculoskelet*)) or ('aromatase inhibitor associated' and (arthralgia or musculoskelet*)) or 'aia' or 'ai' |
| #4 | neurotoxicity or (peripher* and (neuropathy or neuropath* or neurotoxicit*)) or 'cipn' or ('chemotherapy-induced peripheral' and neurotoxicit*) or ('chemotherapy induced peripheral' and neurotoxicit*) or ('chemotherapy-induced peripheral' and neuropath*) or ('chemotherapy induced peripheral' and neuropath*) |
| #5 | chemobrain* or cognitive function impairment or cognitive impair* or cogniti* or neuropsycholog* or acute brain syndrome or acute cerebral insufficiency or acute confusion or acute confusional state or cognitive defect or cognitive dysfunction or cognitive decline or mental deterioration or brain disease or brain disorders |
| #6 | breast neoplasm or ((breast or mamma*) and (cancer* or neopla* or adenocarcin* or carcin* or tumor* or tumour* or malignan* or sarcoma* or mass* or DCIS or ductal* or infiltrat* or intraductal* or lobula* or medullary)) |
| #7 | acupuncture or acupunctur* or acupoint* or acupuncture therap* or electroacupunctur* or electroacupuncture therap* or "zhen jiu" or "zhenjiu" or 'dry needle' |
| #8 | double-blind procedure or randomized controlled trial or single-blind procedure or random* or factorial* or placebo* or doubl* blind* or singl* blind* or assign* or allocat* or volunteer* |
| #9 | (#2 or #3 or #4 or #5) and #1 and #6 and #7 and #8 |

EMBASE

| **ID** | **Search syntax** |
| --- | --- |
| 1 | chemotherapy/ |
| 2 | ('therapy, drug' or 'drug therap*' or 'therapies, drug' or 'chemotherap*' or 'pharmacotherap*' or 'pharmacologic therap*' or chemotherapies or vincristine or oncovin or vinblastine or vinorelbine or vindesine or vinorelbine or vinca alkaloid or vinca alkaloids or oxaliplatin or eloxatin or cisplatin or carboplatin or platinum or platinums or taxane or taxanes or docetaxel or paclitaxel or ifosfamide or ifex or Ixabepilone or epothilone or Epothilones or Bortezomib or proteasome inhibitor or proteasome inhibitors or Thalidomide or Lenalidomide or procarbazine or thiotepa or podophyllin or topoisomerase inhibitor or teniposide or etoposide or vepesid or vumon or gemcitabine or Induction Chemotherapy or Chemotherapy, Adjuvant or Consolidation Chemotherapy or Maintenance Chemotherapy).ti,ab,kw. |
| 3 | nausea/ or vomiting/ |
| 4 | ('nausea' or 'antiemetic*' or 'emetic*' or 'emesis' or vomiting').ti,ab,kw. |
| 5 | (('chemotherapy induced nausea' and 'vomiting') or ('chemotherapy-induced nausea' and 'vomiting') or 'cinv').ti,ab,kw. |
| 6 | arthralgia/ or aromatase inhibitor-associated arthralgia/ |
| 7 | (aromatase adj3 (inhibit* or anastrozole or exemestane or letrozole or aminoglutethimide* or atamestane or formestane or vorozole or arimidex or aromasin or femara or fadrozole or lentaron or rivizor or cytadren)).ti,ab,kw. |
| 8 | (('aromatase inhibitor-associated' adj3 ('arthralgia' or 'musculoskelet*')) or ('aromatase inhibitor associated adj3 ('arthralgia' or 'musculoskelet*')) or 'aia' or 'ai').ti,ab,kw. |
| 9 | neurotoxicity/ |
| 10 | (('peripher*' adj3 ('neuropathy' or 'neuropath*' or 'neurotoxicit*')) or 'cipn').ti,ab,kw. |
| 11 | (('chemotherapy-induced peripheral' and 'neurotoxicit*') or ('chemotherapy induced peripheral' and 'neurotoxicit*') or ('chemotherapy-induced peripheral' and 'neuropath*') or ('chemotherapy induced peripheral' and 'neuropath*')).ti,ab,kw. |
| 12 | chemobrain*.mp. [mp=title, abstract, heading word, drug trade name, original title, device manufacturer, drug manufacturer, device trade name, keyword, floating subheading word, candidate term word] |
| 13 | cognitive function impairment.mp. [mp=title, abstract, heading word, drug trade name, original title, device manufacturer, drug manufacturer, device trade name, keyword, floating subheading word, candidate term word] |
| 14 | ('cognitive impair*' or 'cogniti*' or 'neuropsycholog*' or 'acute brain syndrome' or 'acute cerebral insufficiency' or 'acute confusion' or 'acute confusional state' or 'cognitive defect' or (cognitive adj2 (dysfunction or decline)) or (mental adj2 deterioration) or 'brain disease' or 'brain disorders').ti,ab,kw. |
| 15 | 1 or 2 |
| 16 | 3 or 4 or 5 |
| 17 | 6 or 7 or 8 |
| 18 | 9 or 10 or 11 |
| 19 | 12 or 13 or 14 |
| 20 | breast neoplasm.mp. [mp=title, abstract, heading word, drug trade name, original title, device manufacturer, drug manufacturer, device trade name, keyword, floating subheading word, candidate term word] |
| 21 | ((breast or mamma*) adj3 (cancer* or neopla* or adenocarcin* or carcin* or tumor* or tumour* or malignan* or sarcoma* or mass* or DCIS or ductal* or infiltrat* or intraductal* or lobula* or medullary)).ti,ab,kw. |
| 22 | acupuncture/ |
| 23 | (acupunctur* or acupoint* or acupuncture therap* or electroacupunctur* or electroacupuncture therap* or "zhen jiu" or "zhenjiu" or 'dry needle').ti,ab,kw. |
| 24 | 20 or 21 |
| 25 | 22 or 23 |
| 26 | 15 and 24 and 25 |
| 27 | (16 or 17 or 18 or 19) and 26 |
| 28 | ("double-blind procedure" or "randomized controlled trial" or "single-blind procedure" or random* or factorial* or placebo* or "doubl* blind*" or "singl* blind*" or assign* or allocat* or volunteer*).mp. [mp=title, abstract, heading word, drug trade name, original title, device manufacturer, drug manufacturer, device trade name, keyword, floating subheading word, candidate term word] |
| 29 | 27 and 28 |

CINAHL Plus

| **ID** | **Search syntax** |
| --- | --- |
| S1 | (MH "drug therapy+") |
| S2 | 'therapy, drug' or 'drug therap*' or 'therapies, drug' or 'chemotherap*' or 'pharmacotherap*' or 'pharmacologic therap*' or chemotherapies or vincristine or oncovin or vinblastine or vinorelbine or vindesine or vinorelbine or 'vinca alkaloid' or 'vinca alkaloids' or oxaliplatin or eloxatin or cisplatin or carboplatin or platinum or platinums or taxane or taxanes or docetaxel or paclitaxel or ifosfamide or ifex or Ixabepilone or epothilone or Epothilones or Bortezomib or 'proteasome inhibitor' or 'proteasome inhibitors' or Thalidomide or Lenalidomide or procarbazine or thiotepa or podophyllin or 'topoisomerase inhibitor' or teniposide or etoposide or vepesid or vumon or gemcitabine or 'Induction Chemotherapy' or 'Chemotherapy, Adjuvant' or 'Consolidation Chemotherapy' or 'Maintenance Chemotherapy' |
| S3 | nausea or vomiting |
| S4 | nausea or 'antiemetic*' or 'emetic*' or emesis or vomiting |
| S5 | ('chemotherapy induced nausea' and 'vomiting') or ('chemotherapy-induced nausea' and 'vomiting') or cinv |
| S6 | arthralgia or 'aromatase inhibitor-associated arthralgia' |
| S7 | aromatase N3 (inhibit* or anastrozole or exemestane or letrozole or aminoglutethimide* or atamestane or formestane or vorozole or arimidex or aromasin or femara or fadrozole or lentaron or rivizor or cytadren) |
| S8 | ('aromatase inhibitor-associated' N3 ('arthralgia' or 'musculoskelet*')) or ('aromatase inhibitor associated N3 ('arthralgia' or 'musculoskelet*')) or 'aia' or 'ai' |
| S9 | neurotoxicity |
| S10 | 'peripher*' N3 ('neuropathy' or 'neuropath*' or 'neurotoxicit*') or 'cipn' |
| S11 | (('chemotherapy-induced peripheral' and 'neurotoxicit*') or ('chemotherapy induced peripheral' and 'neurotoxicit*') or ('chemotherapy-induced peripheral' and 'neuropath*') or ('chemotherapy induced peripheral' and 'neuropath*')) |
| S12 | chemobrain* |
| S13 | ‘cognitive function impairment’ |
| S14 | 'cognitive impair*' or 'cogniti*' or 'neuropsycholog*' or 'acute brain syndrome' or 'acute cerebral insufficiency' or 'acute confusion' or 'acute confusional state' or 'cognitive defect' or 'cognitive dysfunction' or 'cognitive decline' or 'mental deterioration' or 'brain disease' or 'brain disorders' |
| S15 | S1 OR S2 |
| S16 | S3 OR S4 OR S5 |
| S17 | S6 OR S7 OR S8 |
| S18 | S9 OR S10 OR S11 |
| S19 | S12 OR S13 OR S14 |
| S20 | breast neoplasm |
| S21 | (breast or mamma*) N3 (cancer* or neopla* or adenocarcin* or carcin* or tumor* or tumour* or malignan* or sarcoma* or mass* or DCIS or ductal* or infiltrat* or intraductal* or lobula* or medullary) |
| S22 | acupuncture |
| S23 | acupunctur* or acupoint* or acupuncture therap* or electroacupunctur* or electroacupuncture therap* or "zhen jiu" or "zhenjiu" or 'dry needle' |
| S24 | S20 OR S21 |
| S25 | S22 OR S23 |
| S26 | S15 AND S24 AND S25 |
| S27 | (S16 OR S17 OR S18 OR S19) AND S26 |
| S28 | "double-blind procedure" or "randomized controlled trial" or "single-blind procedure" or random* or factorial* or placebo* or "doubl* blind*" or "singl* blind*" or assign* or allocat* or volunteer* |
| S29 | S27 AND S28 |

MEDLINE via OVID

| **ID** | **Search syntax** |
| --- | --- |
| 1 | chemotherapy/ |
| 2 | ('therapy, drug' or 'drug therap*' or 'therapies, drug' or 'chemotherap*' or 'pharmacotherap*' or 'pharmacologic therap*' or chemotherapies or vincristine or oncovin or vinblastine or vinorelbine or vindesine or vinorelbine or vinca alkaloid or vinca alkaloids or oxaliplatin or eloxatin or cisplatin or carboplatin or platinum or platinums or taxane or taxanes or docetaxel or paclitaxel or ifosfamide or ifex or Ixabepilone or epothilone or Epothilones or Bortezomib or proteasome inhibitor or proteasome inhibitors or Thalidomide or Lenalidomide or procarbazine or thiotepa or podophyllin or topoisomerase inhibitor or teniposide or etoposide or vepesid or vumon or gemcitabine or Induction Chemotherapy or Chemotherapy, Adjuvant or Consolidation Chemotherapy or Maintenance Chemotherapy).ti,ab,kw. |
| 3 | nausea/ or vomiting/ |
| 4 | ('nausea' or 'antiemetic*' or 'emetic*' or 'emesis' or vomiting').ti,ab,kw. |
| 5 | (('chemotherapy induced nausea' and 'vomiting') or ('chemotherapy-induced nausea' and 'vomiting') or 'cinv').ti,ab,kw. |
| 6 | arthralgia/ or aromatase inhibitor-associated arthralgia/ |
| 7 | (aromatase adj3 (inhibit* or anastrozole or exemestane or letrozole or aminoglutethimide* or atamestane or formestane or vorozole or arimidex or aromasin or femara or fadrozole or lentaron or rivizor or cytadren)).ti,ab,kw. |
| 8 | (('aromatase inhibitor-associated' adj3 ('arthralgia' or 'musculoskelet*')) or ('aromatase inhibitor associated adj3 ('arthralgia' or 'musculoskelet*')) or 'aia' or 'ai').ti,ab,kw. |
| 9 | neurotoxicity/ |
| 10 | (('peripher*' adj3 ('neuropathy' or 'neuropath*' or 'neurotoxicit*')) or 'cipn').ti,ab,kw. |
| 11 | (('chemotherapy-induced peripheral' and 'neurotoxicit*') or ('chemotherapy induced peripheral' and 'neurotoxicit*') or ('chemotherapy-induced peripheral' and 'neuropath*') or ('chemotherapy induced peripheral' and 'neuropath*')).ti,ab,kw. |
| 12 | chemobrain*.mp. [mp=title, abstract, heading word, drug trade name, original title, device manufacturer, drug manufacturer, device trade name, keyword, floating subheading word, candidate term word] |
| 13 | cognitive function impairment.mp. [mp=title, abstract, heading word, drug trade name, original title, device manufacturer, drug manufacturer, device trade name, keyword, floating subheading word, candidate term word] |
| 14 | ('cognitive impair*' or 'cogniti*' or 'neuropsycholog*' or 'acute brain syndrome' or 'acute cerebral insufficiency' or 'acute confusion' or 'acute confusional state' or 'cognitive defect' or (cognitive adj2 (dysfunction or decline)) or (mental adj2 deterioration) or 'brain disease' or 'brain disorders').ti,ab,kw. |
| 15 | 1 or 2 |
| 16 | 3 or 4 or 5 |
| 17 | 6 or 7 or 8 |
| 18 | 9 or 10 or 11 |
| 19 | 12 or 13 or 14 |
| 20 | breast neoplasm.mp. [mp=title, abstract, heading word, drug trade name, original title, device manufacturer, drug manufacturer, device trade name, keyword, floating subheading word, candidate term word] |
| 21 | ((breast or mamma*) adj3 (cancer* or neopla* or adenocarcin* or carcin* or tumor* or tumour* or malignan* or sarcoma* or mass* or DCIS or ductal* or infiltrat* or intraductal* or lobula* or medullary)).ti,ab,kw. |
| 22 | acupuncture/ |
| 23 | (acupunctur* or acupoint* or acupuncture therap* or electroacupunctur* or electroacupuncture therap* or "zhen jiu" or "zhenjiu" or 'dry needle').ti,ab,kw. |
| 24 | 20 or 21 |
| 25 | 22 or 23 |
| 26 | 15 and 24 and 25 |
| 27 | (16 or 17 or 18 or 19) and 26 |
| 28 | ("double-blind procedure" or "randomized controlled trial" or "single-blind procedure" or random* or factorial* or placebo* or "doubl* blind*" or "singl* blind*" or assign* or allocat* or volunteer*).mp. [mp=title, abstract, heading word, drug trade name, original title, device manufacturer, drug manufacturer, device trade name, keyword, floating subheading word, candidate term word] |
| 29 | 27 and 28 |

AMED

| **ID** | **Search syntax** |
| --- | --- |
| 1 | (drug therapy or 'therapy, drug' or drug therap* or 'therapies, drug' or chemotherap* or pharmacotherap* or pharmacologic therap* or chemotherapies or vincristine or oncovin or vinblastine or vinorelbine or vindesine or vinorelbine or 'vinca alkaloid' or 'vinca alkaloids' or oxaliplatin or eloxatin or cisplatin or carboplatin or platinum or platinums or taxane or taxanes or docetaxel or paclitaxel or ifosfamide or ifex or Ixabepilone or epothilone or Epothilones or Bortezomib or 'proteasome inhibitor' or 'proteasome inhibitors' or Thalidomide or Lenalidomide or procarbazine or thiotepa or podophyllin or 'topoisomerase inhibitor' or teniposide or etoposide or vepesid or vumon or gemcitabine or 'Induction Chemotherapy' or 'Chemotherapy, Adjuvant' or 'Consolidation Chemotherapy' or 'Maintenance Chemotherapy').mp. [mp=abstract, heading words, title] (29556) |
| 2 | (nausea or vomiting or antiemetic* or emetic* or emesis or ('chemotherapy induced nausea' and 'vomiting') or ('chemotherapy-induced nausea' and 'vomiting') or cinv).mp. [mp=abstract, heading words, title] (1166) |
| 3 | (arthralgia or 'aromatase inhibitor-associated arthralgia' or (aromatase and (inhibit* or anastrozole or exemestane or letrozole or aminoglutethimide* or atamestane or formestane or vorozole or arimidex or aromasin or femara or fadrozole or lentaron or rivizor or cytadren)) or ('aromatase inhibitor-associated' and (arthralgia or musculoskelet*)) or ('aromatase inhibitor associated' and (arthralgia or musculoskelet*)) or 'aia' or 'ai').mp. [mp=abstract, heading words, title] (455) |
| 4 | (neurotoxicity or (peripher* and (neuropathy or neuropath* or neurotoxicit*)) or 'cipn' or ('chemotherapy-induced peripheral' and neurotoxicit*) or ('chemotherapy induced peripheral' and neurotoxicit*) or ('chemotherapy-induced peripheral' and neuropath*) or ('chemotherapy induced peripheral' and neuropath*)).mp. [mp=abstract, heading words, title] (923) |
| 5 | (chemobrain* or cognitive function impairment or cognitive impair* or cogniti* or neuropsycholog* or acute brain syndrome or acute cerebral insufficiency or acute confusion or acute confusional state or cognitive defect or cognitive dysfunction or cognitive decline or mental deterioration or brain disease or brain disorders).mp. [mp=abstract, heading words, title] (12558) |
| 6 | (breast neoplasm or ((breast or mamma*) and (cancer* or neopla* or adenocarcin* or carcin* or tumor* or tumour* or malignan* or sarcoma* or mass* or DCIS or ductal* or infiltrat* or intraductal* or lobula* or medullary))).mp. [mp=abstract, heading words, title] (2506) |
| 7 | (acupuncture or acupunctur* or acupoint* or acupuncture therap* or electroacupunctur* or electroacupuncture therap* or "zhen jiu" or "zhenjiu" or 'dry needle').mp. [mp=abstract, heading words, title] (11179) |
| 8 | (double-blind procedure or randomized controlled trial or single-blind procedure or random* or factorial* or placebo* or doubl* blind* or singl* blind* or assign* or allocat* or volunteer*).mp. [mp=abstract, heading words, title] (28198) |
| 9 | (2 or 3 or 4 or 5) and 1 and 6 and 7 and 8 (5) |

CNKI

| (SU=('噁心'+'嘔吐'+'關節炎'+'痹證'+'關節痛'+'神經毒性'+'神經病變'+'認知'+'腦損傷'+'記憶' ) AND (SU=('乳癌'+'乳腺癌'+'乳腺惡性腫瘤') AND (SU=('針'+'針刺'+'電針'+'腹針'+'耳針') AND (SU='臨床' OR TI='臨床' OR KY='臨床') AND (SU='化療'+'化學藥物'+'化學療法'+'環磷醯胺'+'氨甲蝶呤' +'5-氟尿嘧啶'+'阿黴素'+'環磷醯胺'+'順鉑') |
| --- |
| *Translated in English: (SU=('Nausea'+'Vomiting'+'Arthritis'+'Arthritis'+'Arthralgia'+'Neurotoxicity'+'Neuropathy'+'Cognition'+'Brain Injury'+'Memory') AND (SU=('breast cancer'+'breast cancer'+'breast malignant tumor') AND (SU=('needle'+'acupuncture'+'electroacupuncture'+'abdominal acupuncture'+'ear acupuncture') AND (SU='clinical' OR TI='clinical' OR KY='clinical') AND (SU='chemotherapy'+'chemotherapy'+'chemotherapy'+'cyclophosphamide'+'methotrexate' +'5-Fluorouracil'+'Adriamycin'+'Cyclophosphamide'+'Cisplatin')* |

Wanfang Database

| 摘要:('惡心'+'嘔吐'+'關節炎'+'痹證'+'關節痛'+'神經毒性'+'神經病變'+'認知'+'腦損傷'+'記憶' ) AND 摘要:('乳癌'+'乳腺癌'+'乳腺惡性腫瘤') AND 摘要:('針'+'針刺'+'電針'+'腹針'+'耳針') AND 摘要:('臨床') AND 摘要:('化療'+'化學藥物'+'化學療法'+'環磷醯胺'+'氨甲蝶呤' +'5-氟尿嘧啶'+'阿黴素'+'環磷醯胺'+'順鉑') |
| --- |
| *Translated in English:*  *Abstract: ('Nausea'+'vomiting'+'arthritis'+'Arthritis'+'arthralgia'+'neurotoxicity'+'neuropathy'+'cognition'+'brain injury'+'memory') AND abstract: ('breast cancer'+'breast cancer'+'breast malignant tumor') AND abstract: ('needle'+'acupuncture'+'electroacupuncture'+'abdominal acupuncture'+'ear acupuncture') AND abstract: ('Clinical') AND Abstract: ('Chemotherapy'+'Chemical drugs'+'Chemotherapy'+'Cyclophosphamide'+'Methotrexate' +'5-Fluorouracil'+'Adriamycin'+' Cyclophosphamide'+'cisplatin')* |
